# Acute Gastric Dilatation in a 16-Year-Old Patient With Duchenne Muscular Dystrophy: A Case Report and an Updated Literature Review

**DOI:** 10.7759/cureus.96639

**Published:** 2025-11-12

**Authors:** Camilo Novoa Garnica, Carlos J Ruíz Hernandez, Sandra Montells, Maria Homez Arias, Francisco J Martin Carpi

**Affiliations:** 1 Department of Pediatric Gastroenterology, Hospital Sant Joan de Déu, Barcelona, ESP

**Keywords:** acute gastric dilatation, duchenne muscular dystrophy (dmd), gastrointestinal motility, gastrojejunostomy, gastroparesis symptoms

## Abstract

Duchenne muscular dystrophy (DMD) is a progressive neuromuscular disorder associated with degeneration of both skeletal and smooth muscles. Gastrointestinal (GI) complications, including gastroparesis and gastric dilatation, are underrecognized but potentially life-threatening.

We present the case of a 16-year-old patient with DMD who developed acute gastric dilatation secondary to gastroparesis. Imaging revealed severe gastric dilatation and organoaxial gastric volvulus. The patient required nasogastric decompression, prokinetic therapy, endoscopic transpyloric tube placement, and eventually gastrojejunostomy for enteral feeding. After 15 months of nutritional and pharmacologic support, the patient transitioned to gastric and later oral feeding with good tolerance.

GI manifestations in DMD stem from smooth muscle atrophy, altered motility, and delayed gastric emptying. The literature review identified similar cases with varying presentations and treatments. Gastroparesis may be compounded with constipation, increasing the risk of complications.

DMD patients may present with acute gastric dilatation as a consequence of progressive GI dysfunction. Early recognition and intervention with decompression, prokinetics, and enteral support are critical to improve outcomes.

## Introduction

Duchenne muscular dystrophy (DMD) is a progressive X-linked recessive disorder caused by mutations in the dystrophin gene, affecting approximately one in 3,500-5,000 live male births worldwide [1]. It is characterized by progressive skeletal muscle weakness, loss of ambulation during early adolescence, respiratory insufficiency, and cardiomyopathy, which remain the leading causes of morbidity and mortality [1]. Advances in multidisciplinary care have extended life expectancy, but systemic complications continue to pose significant clinical challenges.

Although skeletal muscle involvement is well documented, the impact of DMD on smooth muscle, particularly in the gastrointestinal (GI) tract, is less understood. Pathological changes in GI smooth muscle may cause motility disorders such as delayed gastric emptying, chronic constipation, and acute gastric or intestinal dilatation [1-13]. Such complications are relatively rare but can be life-threatening if not promptly recognized, potentially resulting in dehydration, electrolyte imbalances, acid-base disturbances, or GI perforation [3,5,7-9]. Several studies have reported gastric hypomotility and gastroparesis in DMD, emphasizing the need for careful monitoring of GI symptoms [8-12,14].

Despite the clinical significance, the literature describing acute GI complications in DMD remains limited, and there are no widely accepted management guidelines [6,15]. Most evidence derives from isolated case reports or small series, highlighting the importance of clinical documentation and literature synthesis.

In this context, we report the case of a 16-year-old patient with DMD who developed acute gastric dilatation. We emphasize the importance of early recognition, diagnostic evaluation, therapeutic management, and nutritional support, and we provide an updated review of previously reported cases to place this complication within the broader spectrum of GI involvement in DMD.

A literature review was conducted to identify previously reported cases of acute gastric dilatation or gastroparesis associated with DMD. The PubMed, Scopus, Ovid, and Latin America and the Caribbean Literature on Health Sciences (LILACS) databases were searched using the keywords "Duchenne Muscular Dystrophy,” “Gastric Dilatation," "Gastroparesis," and "Muscular Dystrophy." Reference lists of relevant articles were also reviewed to identify additional reports. Case reports, case series, and original research articles describing GI complications in patients with DMD were considered eligible for inclusion.

## Case presentation

A 16-year-old male patient with a history of DMD, scoliosis, osteoporosis, mild cardiomyopathy, oropharyngeal dysphagia, chronic constipation, hypercalciuria with right renal lithiasis, and hyperuricosuria presented to the emergency department with epigastric pain, bronchial hypersecretion, elevated blood pressure (129/91 mmHg), a heart rate of 70 beats per minute, and oxygen saturation of 96%.

On physical examination, the patient was in a non-ambulatory stage and wheelchair-dependent. He presented with severe scoliosis and marked contractures involving all four limbs. Head control was preserved, although only minimal wrist flexion was observed. There was evident proximal, distal, and truncal muscle atrophy consistent with advanced disease progression.

Abdominal examination showed distension predominantly in the upper hemiabdomen, which was tympanic and soft to palpation, with no signs of peritoneal irritation. Anthropometric evaluation demonstrated chronic moderate malnutrition according to World Health Organization (WHO) reference standards [16] (height: 152.9 cm, ≈ −2.3 SD, P2; weight: 30 kg, ≈ −4.0 SD, < P0.01; BMI: 12.9, ≈ −3.6 SD, < P0.1).

**Figure 1:**
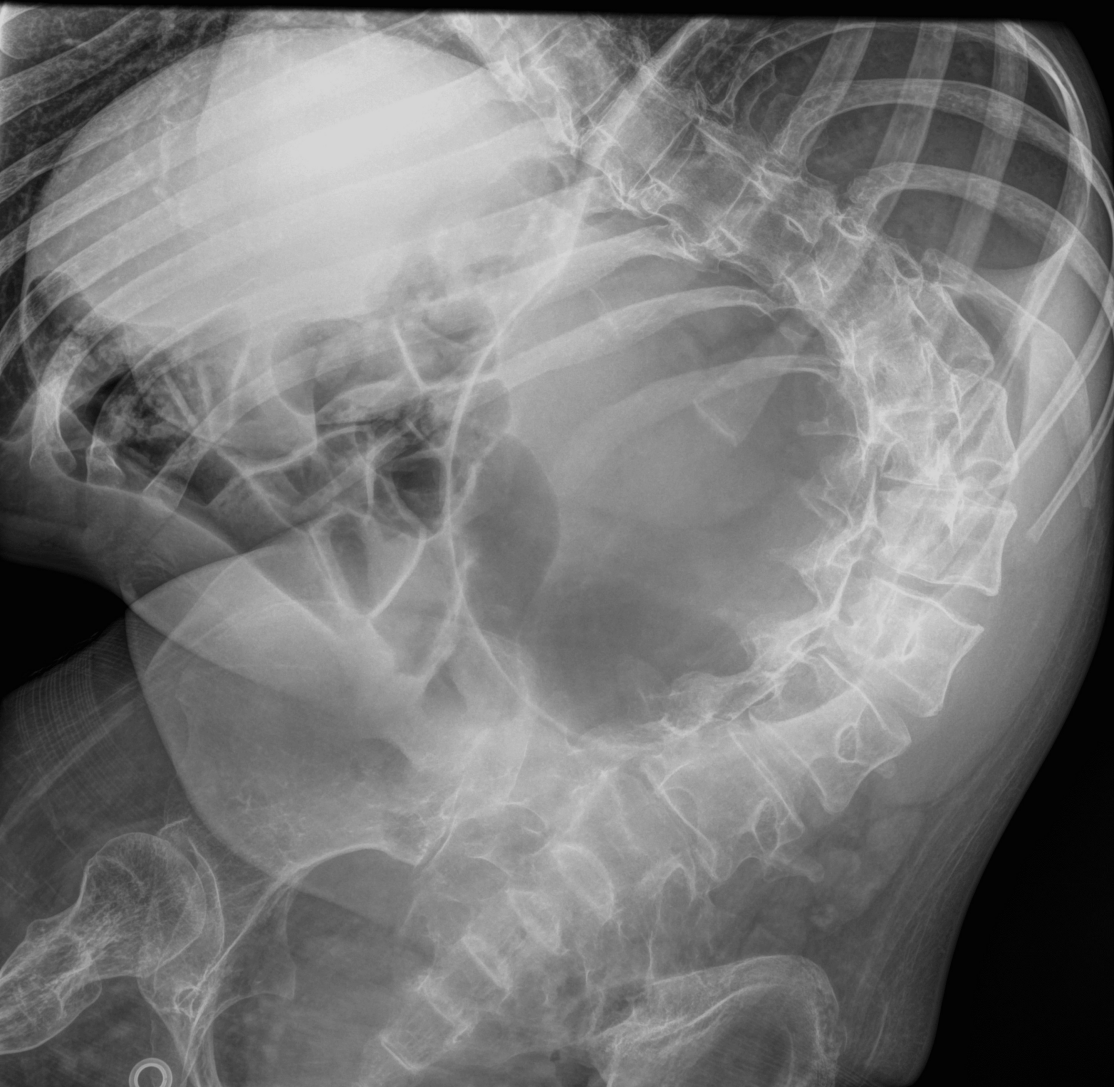
A simple abdominal X-ray shows significant abdominal distension.

Abdominal radiography (Figure 1) demonstrated significant gastric distension, which was considered the cause of the epigastric pain. A nasogastric decompression tube was placed, and oral paracetamol was administered.

Laboratory findings at admission and after 24 hours are summarized in Table 1. The results demonstrated leukocytosis and neutrophilia, along with mild elevations in liver and pancreatic enzymes. The patient was kept nil by mouth and admitted for inpatient management with analgesia, proton pump inhibitors for gastric protection, and intravenous fluids.

**Table 1:** Laboratory findings at admission and after 24 hours WBC: white blood count; AST: aspartate aminotransferase; ALT: alanine aminotransferase; GGT: gamma-glutamyl transpeptidase

Laboratory Test	Reference Range	Initial Value	Value at 24 Hours	Interpretation
WBC (×10³/µL)	4.0–10.0	17.7	13.1	Leukocytosis improving
Neutrophils (×10³/µL)	1.5–5.0	15.2	10.7	Neutrophilia decreasing
AST (IU/L)	<35	67	39	Mildly elevated, decreasing
ALT (IU/L)	<35	96	83	Elevated, slight improvement
GGT (IU/L)	0–50	44	44	Within normal limits at 24 hours and 48 hours
Amylase (IU/L)	60–180	283	235	Elevated, improving
Lipase (IU/L)	<160	286	34	Elevated initially, normalized

**Figure 2:**
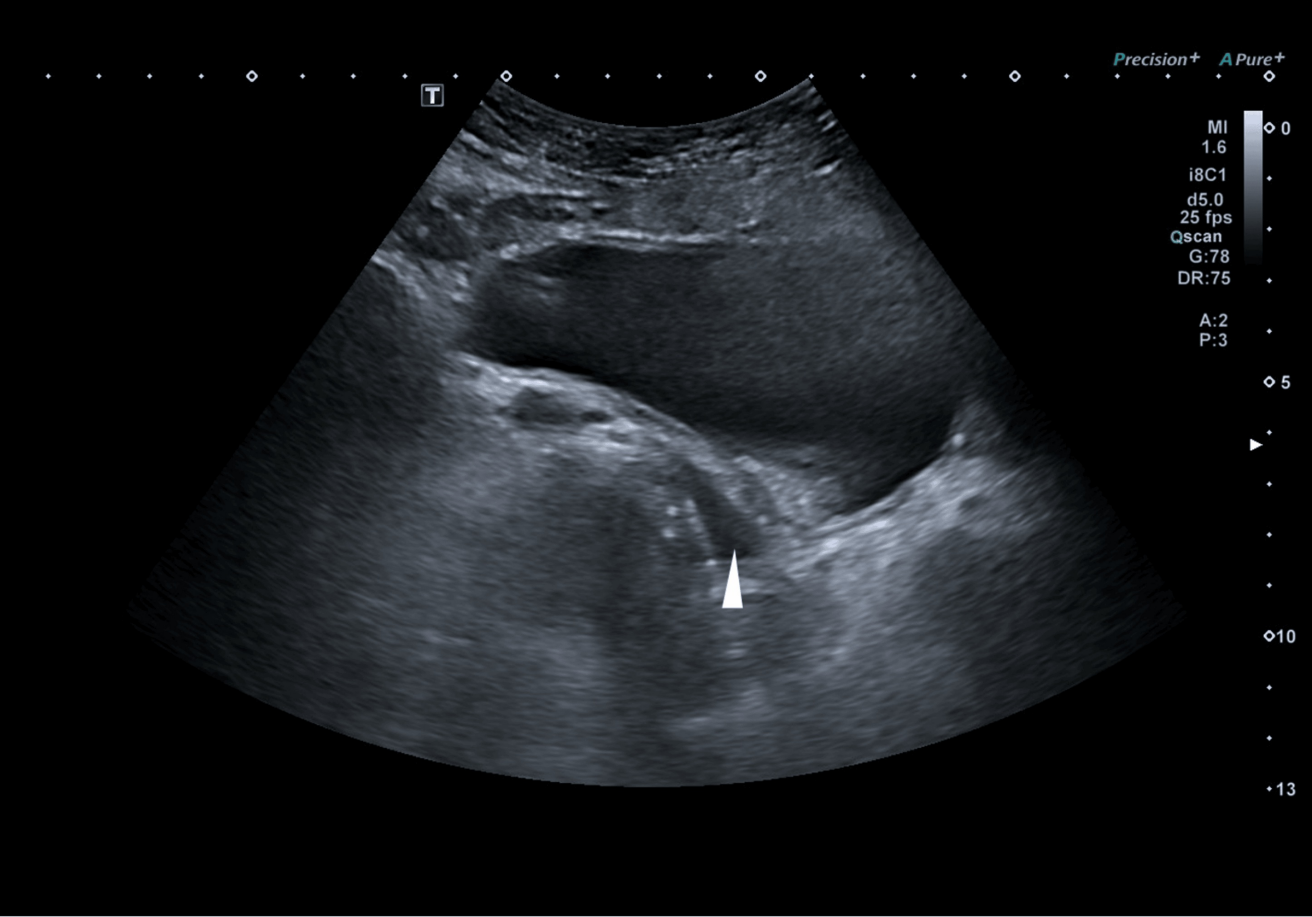
The abdominal ultrasound shows fluid collection, predominantly an anechoic portion of the large image occupying the abdomen, and a well-defined interface between the superior anechoic fluid and the denser, echogenic content (white arrow).

After 24 hours, there was an improvement in pancreatic enzymes and a decrease in liver enzymes. The nasogastric tube was removed; however, the patient reported worsening abdominal pain unresponsive to analgesics. Physical examination revealed marked abdominal distension and intense pain upon superficial palpation. An abdominal ultrasound (Figure 2) was requested, which revealed a large amount of fluid hindering adequate visualization of the pancreas.

**Figure 3:**
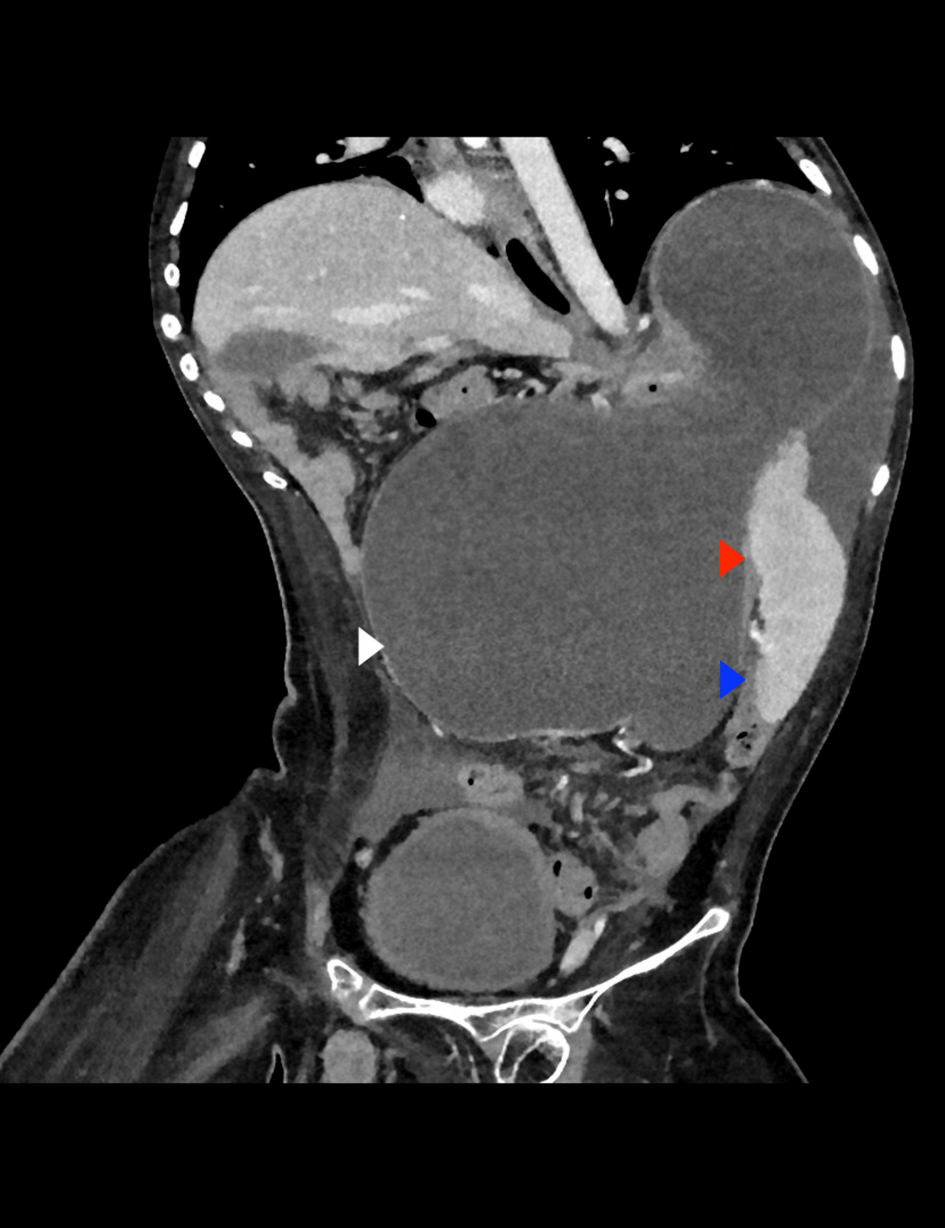
Computed tomography (CT) of the abdomen (coronal view) Severe gastric distention (white arrow) with a large fluid-air level (red arrow), a finding consistent with gastric outlet obstruction, is noted. A small amount of free fluid (blue arrow) is noted in the left peritoneal cavity. This finding is highly suggestive of gastric volvulus.

**Figure 4:**
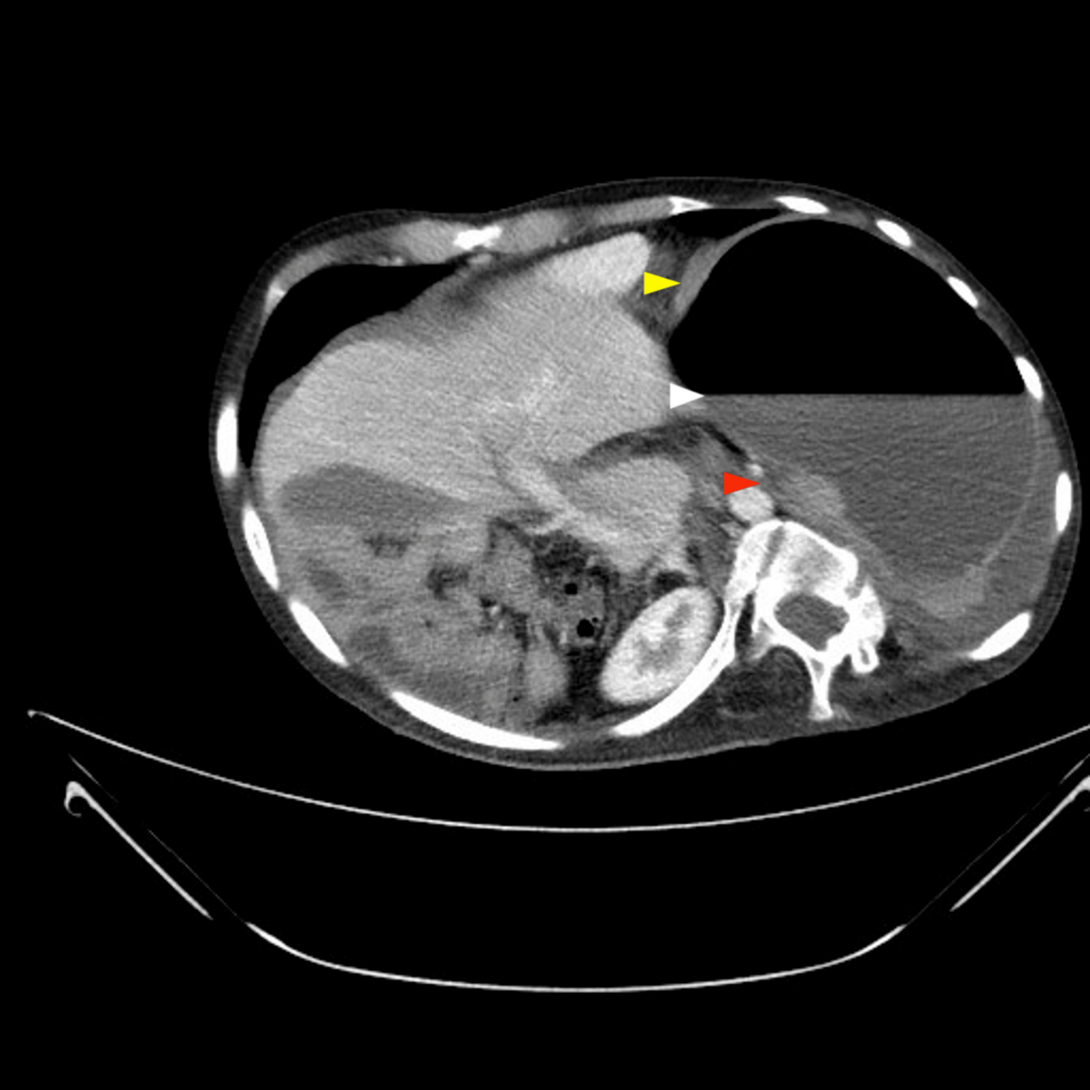
Computed tomography (CT) of the abdomen (axial view) Evident marked gastric dilation with a prominent fluid-air level (white arrow). The image shows the point of torsion (red arrow) of the gastric axis in the posterior-medial region, suggesting the whirlpool sign or a narrowing of the gastroesophageal junction. Additionally, gastric wall thickening (yellow arrow) is observed, consistent with congestion and potential ischemia.

**Figure 5:**
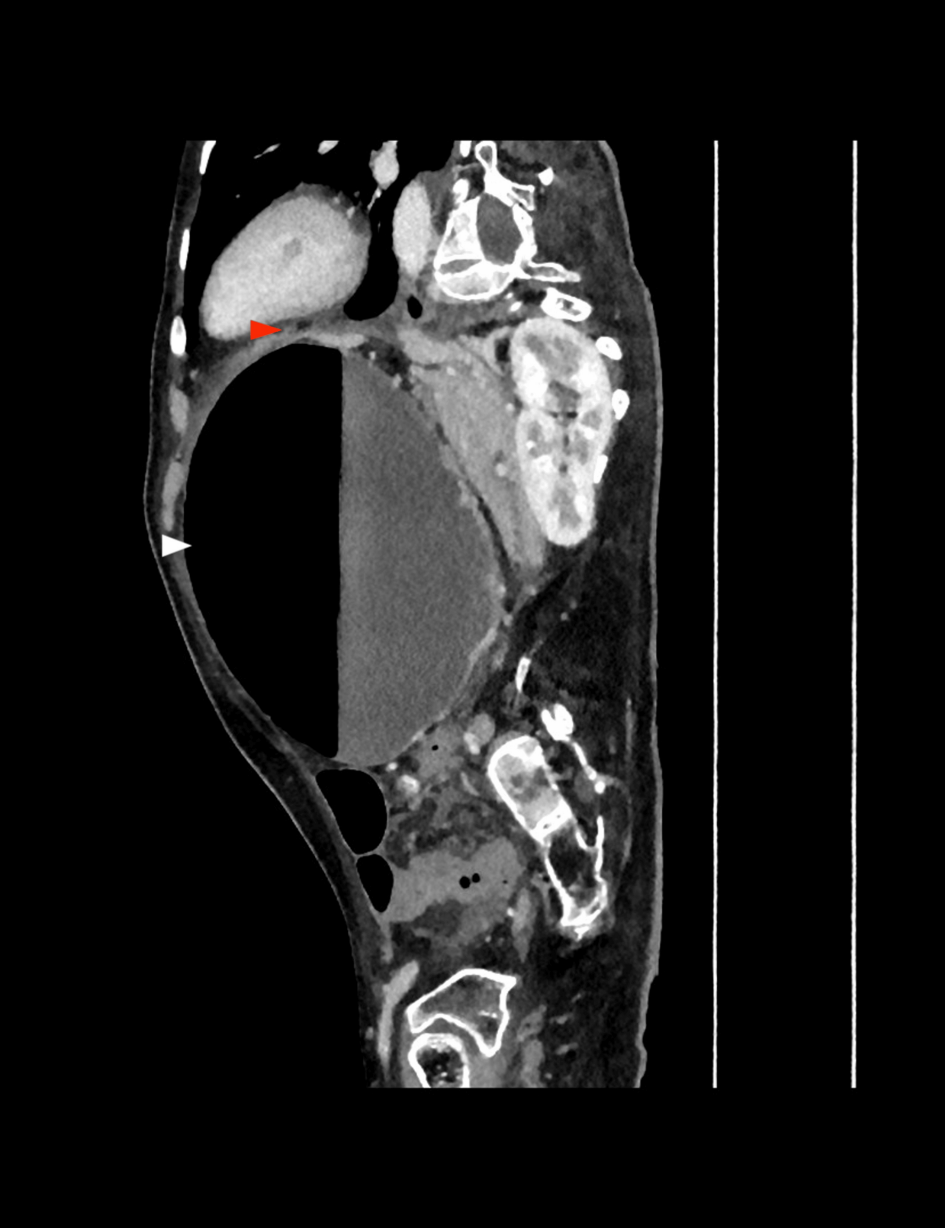
Computed tomography (CT) of the abdomen (sagittal view) Lateral view illustrating the extreme dilation and abnormal positioning of the stomach (white arrow). This projection helps identify gastric inversion (red arrow) along the craniocaudal axis, a key finding of organoaxial gastric volvulus.

A contrast-enhanced abdominal CT scan was performed (Figures 3-5), revealing organoaxial gastric volvulus.

The nasogastric decompression tube was reinserted, draining 1500 mL of fluid and air, with marked improvement in abdominal pain following decompression. Due to suspected delayed gastric emptying secondary to persistent gastric dilatation, intravenous prokinetic therapy was initiated (erythromycin 3 mg/kg every eight hours).

**Figure 6:**
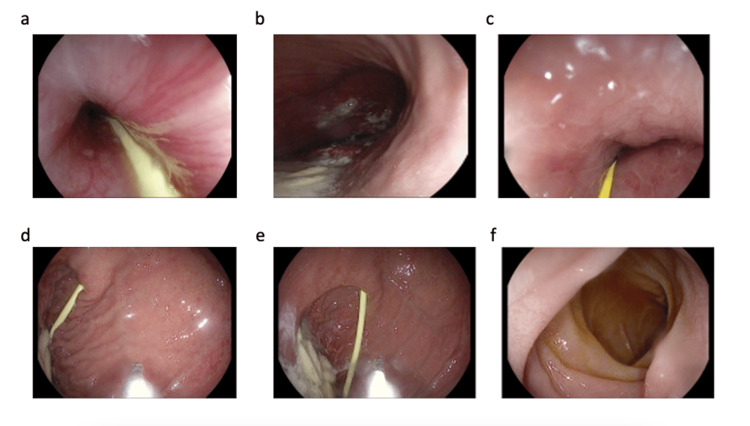
Upper gastrointestinal endoscopy a. Esophagus; b. Gastric body; c. Stomach; d. Transpyloric feeding tube; e. Transpyloric feeding tube; f. Second part of the duodenum

Following improvement in gastric dilatation, a liquid diet was introduced on the seventh day of hospitalization. However, the patient again developed abdominal distension and gastric retention. Due to digestive intolerance, a transpyloric tube was placed via endoscopy under suspicion of gastroparesis or delayed gastric emptying (Figure 6). Endoscopic findings included severe gastric dilatation with abundant fluid content, erosive gastritis, and marked pyloric angulation that hindered localization and access. Despite these challenges, the enteral tube was successfully advanced and positioned in the second portion of the duodenum.

After a new episode of abdominal pain and distension related to feeding, and absence of bowel movements, intravenous pyridostigmine treatment was initiated (initially at 0.6 mg/kg/day, increased to 0.9 mg/kg/day), administered twice daily (every 12 hours).

After 11 days of pyridostigmine treatment, the patient showed favorable progression, tolerating the gradual increase in enteral nutrition via the transpyloric tube until reaching basal caloric and fluid requirements, along with small amounts of water orally.

Given the anticipated need for prolonged use of the transpyloric tube, a gastrojejunostomy tube was placed for decompression and feeding purposes.

During outpatient follow-up, the patient maintained adequate tolerance to jejunal port enteral nutrition. After 11 months, a gradual transition to gastric port feeding was initiated. The patient achieved good weight gain and maintained a regular bowel pattern while continuing on osmotic laxative therapy (polyethylene glycol 0.5 g/kg/day enterally) and prokinetic medications (oral pyridostigmine 0.6 mg/kg/day and erythromycin 3 mg/kg/dose every 8 hours).

At 15 months post-gastrojejunostomy placement, after demonstrating adequate and complete tolerance via the gastric port, the device was switched to a gastrostomy tube. The patient transitioned to enteral bolus feeding and later to oral intake of pureed foods with good tolerance. He is currently maintained on oral pyridostigmine and erythromycin at the discharge-prescribed doses.

## Discussion

DMD is a progressive neuromuscular, X-linked, recessive genetic disorder. Its prevalence in the general population is approximately one in 5,000 live-born male infants. The DMD gene encodes the dystrophin protein, located on the short arm of the X chromosome in region p21. Various mutations in this gene lead to progressive muscle damage, resulting in muscular weakness, loss of ambulation, respiratory deterioration, and cardiomyopathy [1]. Although GI involvement has been less extensively studied, smooth muscle atrophy and degeneration are well-documented and may lead to severe clinical manifestations such as gastric dilatation, as in our patient [2]. These manifestations contribute to increased morbidity and mortality in this population [3].

The true frequency of gastric dilatation among individuals with DMD has not been clearly established. Historical reports dating back to 1945 have documented patients with muscular dystrophy showing GI tract abnormalities, including clinically observed gastric dilatation [3]. Experimental studies using animal models have also shown that the lack of dystrophin results in pathological alterations of the GI system, leading to reduced smooth muscle contractility and impaired peristaltic activity [1]. These findings may account for the high prevalence of GI symptoms reported in DMD, ranging from constipation and abdominal distension to severe gastroparesis.

Our literature review identified only a limited number of published cases of gastric dilatation associated with DMD (Table 2). The majority of the patients described were adolescents or young adults presenting with abdominal pain, nausea, vomiting, or constipation, with radiographic evidence of gastric or intestinal dilatation [4,5]. Despite heterogeneity in presentation, these reports consistently suggest that GI motor dysfunction is an underrecognized but clinically significant manifestation of DMD. Most published cases, including those described by Bevans [4] and Crowe [5], report predominant involvement of the upper GI tract, particularly the stomach and small intestine, manifesting as gastric dilatation, delayed gastric emptying, or small bowel dysmotility, whereas colonic involvement appears to be less frequent. Table 2 compiles case reports and studies identified through a literature search conducted in various scientific databases (PubMed, Ovid, Scopus, and LILACS) using the keywords “Duchenne Muscular Dystrophy,” “Gastric Dilatation,” “Gastroparesis,” and “Muscular Dystrophy,” along with manual searches. A total of 12 articles that met the inclusion criteria were reviewed in full.

**Table 2:** Case reports and studies on gastric dilatation and DMD described in the literature NR: not reported; PMD: progressive muscular dystrophy; DMD: Duchenne muscular dystrophy; GI: gastrointestinal tract; X-RAY: radiography; LEV: intravenous fluids; NG tube: nasogastric tube; TEGD: esophagogastroduodenal transit; CO₂: carbon dioxide

Reference	Number of Patients	Year	Age (Years)	Disease	Symptoms	Diagnostic studies	Treatment
Dhaliwal A et al. [2]	1	2019	21	DMD	Constipation, vomiting, abdominal pain	Abdominal CT scan	IV fluids, NG tube
Bensen et al. [3]	1	1996	19	DMD	Severe epigastric pain, nausea, anorexia, oliguria	Abdominal X-ray	NG tube, IV fluids, broad-spectrum antibiotics, oral ranitidine, cisapride, ursodeoxycholic acid
Bevans [4]	4	1945	NR	PMD	Four autopsies showing GI tract histopathology abnormalities; two with marked gastric dilatation; one with gastric perforation	NR	NR
Crowe et al. [5]	1	1961	9	Pelvifemoral girdle muscular dystrophy	Odynophagia, generalized abdominal pain, vomiting	Abdominal X-ray	IV fluids, blood transfusion
Robin et al. [6]	14	1963	Multiple ages reported between five and 15.	PMD	General malaise, abdominal pain, 'coffee-ground' vomiting, fever	NR	Gastric lavage and aspiration via NG tube, IV fluids
Satrk et al. [7]	1	1988	26	DMD	Nausea, bilious vomiting, chronic constipation with fecal impaction	Abdominal X-ray, Upper GI series	IV fluids, NG tube
Barohn et al. [8]	12	1988	Multiple ages reported between 13 and 27 years	DMD	Epigastric pain, vomiting, abdominal distension, dyspnea	Abdominal X-ray, gastric emptying scan	NG aspiration, NR
Staiano et al. [9]	15	1992	Multiple ages reported between three and 19.	PMD	Anorexia, constipation	Gastric emptying scintigraphy, esophageal manometry	NR
Rohira et al. [10]	1	1993	36	PMD	Nausea and vomiting	Gastric emptying scintigraphy	NR
Chung et al. [11]	1	1998	14	DMD	Vomiting, abdominal pain, anorexia, constipation, marked abdominal distension, absent bowel sounds, tympanic abdomen	Abdominal X-ray, cutaneous electrogastrography	Erythromycin, metoclopramide
Lunshof et al. [12]	1	2000	15	DMD	Nausea, bilious vomiting, abdominal pain	Abdominal X-ray	IV fluids, NG tube
Lo Cascio et al. [13]	33	2016	Multiple ages reported between 12 and 41.	DMD	Stomach fullness, loss of appetite, inability to finish meals, abdominal distension, nausea, vomiting, constipation	CO₂ exhalation after ¹³C-labeled meals, abdominal X-ray	NR

In a case-control study, Jaffe et al. [14] compared 55 individuals with DMD to healthy participants of similar age. The DMD group showed a higher frequency of swallowing difficulties, nasal resonance, choking episodes during meals, throat clearing after eating, heartburn, and postprandial vomiting. These manifestations tended to be more common in older, non-ambulatory patients than in younger, ambulatory children with DMD.

Another GI condition commonly seen in DMD patients is gastroparesis, which leads to GI dilation. Several publications have shown that gastric emptying times in DMD patients of various ages are significantly prolonged [2]. This occurs due to impaired smooth muscle contractility and autonomic dysfunction secondary to dystrophin deficiency, resulting in delayed gastric emptying and progressive gastric distension, which may occasionally extend to the proximal small intestine. In a comparative study conducted by Barohn et al. [8], 11 patients with DMD aged 11 to 27 were compared to healthy controls using radionuclide scintigraphy. The study showed that gastric emptying in DMD subjects was significantly delayed compared to the eleven healthy controls. More recently, Lo Cascio et al. [13] evaluated 33 DMD patients aged 12 to 41 years. They assessed dietary behavior and GI symptoms using questionnaires and performed tests such as the ¹³C gastric emptying breath test (measurement of carbon dioxide (CO₂) breath curves after ingestion of ¹³C-labeled meals) to evaluate gastric emptying and abdominal X-rays following the administration of radiopaque (Sitz) markers to assess colonic transit time. They concluded that DMD patients have significantly altered GI motor function, and since objective measures of GI transport impairment did not correlate with gastroparesis or constipation symptoms, objective testing should always be performed, whenever possible, to ensure proper intestinal transit, regardless of the patient’s symptom perception, to prevent life-threatening complications [14].

Our patient presented with severe gastric dilatation associated with abdominal pain and constipation, manifestations consistent with previously reported cases. The clinical course highlights the importance of recognizing GI complications in DMD, as delayed diagnosis may lead to life-threatening complications.

Despite the growing recognition of GI manifestations in DMD, there are still no standardized guidelines for their management [2]. The 2014 DMD Care Considerations Update emphasized the need for multidisciplinary approaches, including dietary modifications, pharmacological treatment with prokinetics, and postpyloric feeding when necessary. Evidence suggests that prokinetic agents may improve symptoms and GI motility in selected cases [2-15], though systematic studies are lacking.

Overall, our case underscores two critical points: first, that GI involvement in DMD is more common than generally perceived and may present with acute, potentially life-threatening complications such as gastric dilatation; and second, that proactive screening and timely initiation of supportive measures may improve outcomes. Future research should focus on defining standardized protocols for diagnosis and treatment, thereby reducing morbidity and mortality in this vulnerable patient population.

## Conclusions

GI manifestations in DMD are common but often underdiagnosed. Acute gastric dilatation, although rare, represents a potentially life-threatening complication that requires prompt recognition and treatment. This case illustrates that a stepwise, multidisciplinary approach, including nasogastric decompression, prokinetic therapy, and postpyloric feeding, can lead to favorable long-term outcomes, even allowing eventual recovery of gastric and oral tolerance.

Healthcare providers caring for patients with DMD should maintain a high index of suspicion for GI dysfunction, especially in the presence of abdominal pain, distension, or feeding intolerance. Early diagnosis and timely intervention are key to preventing complications, reducing morbidity, and improving quality of life.
